# Control of Immunoregulatory Molecules by miRNAs in T Cell Activation

**DOI:** 10.3389/fimmu.2018.02148

**Published:** 2018-09-25

**Authors:** Ana Rodríguez-Galán, Lola Fernández-Messina, Francisco Sánchez-Madrid

**Affiliations:** ^1^Servicio de Inmunología, Instituto de Investigación Sanitaria Princesa (IP), Hospital Universitario de la Princesa, Universidad Autónoma de Madrid, Madrid, Spain; ^2^Centro Nacional de Investigaciones Cardiovasculares, Madrid, Spain; ^3^Centro de Investigación Biomédica en Red de Enfermedades Cardiovasculares, Madrid, Spain

**Keywords:** T cell activation, microRNAs (miRNAs), immunoregulatory molecules, miRNA signature, CD4, CD8, T lymphocyte

## Abstract

MiRNA targeting of key immunoregulatory molecules fine-tunes the immune response. This mechanism boosts or dampens immune functions to preserve homeostasis while supporting the full development of effector functions. MiRNA expression changes during T cell activation, highlighting that their function is constrained by a specific spatiotemporal frame related to the signals that induce T cell-based effector functions. Here, we update the state of the art regarding the miRNAs that are differentially expressed during T cell stimulation. We also revisit the existing data on miRNA function in T cell activation, with a special focus on the modulation of the most relevant immunoregulatory molecules.

## Introduction

MiRNAs are small (~19–24 nucleotides) single-stranded non-coding RNA species that act as post-transcriptional modulators; they control gene expression, either by promoting mRNAs degradation or repressing their translation (1). More than 2,500 human mature miRNA sequences have been already listed in MirBase (2) although the total amount of miRNAs is likely up to 10 times higher (3). Friedman et al. (4) estimated that miRNAs could modulate around 60% of protein-coding genes, indicating the relevance of these regulatory pathways in gene expression.

**Figure 1:**
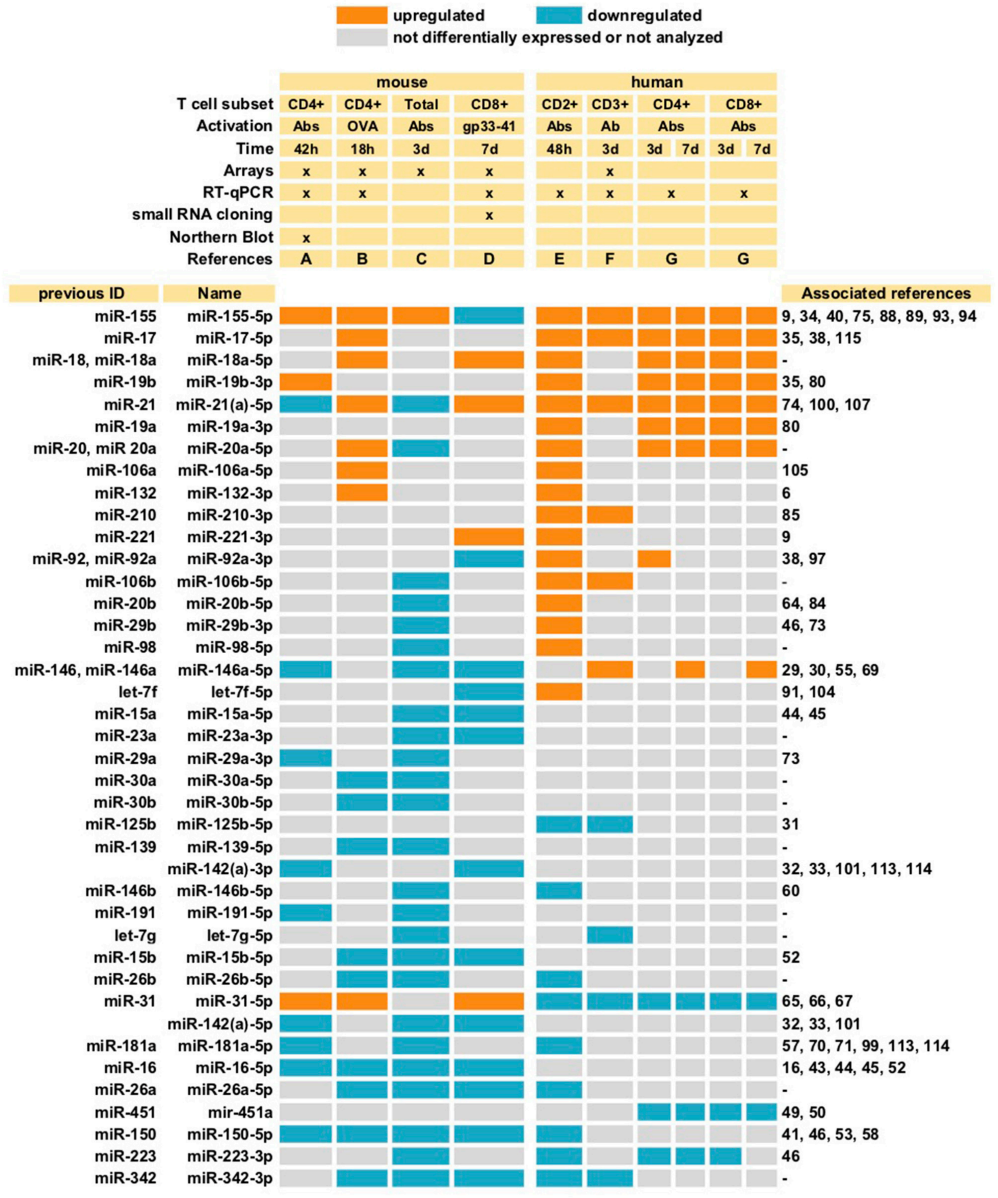
MiRNAs differentially expressed upon T cell stimulation. MiRNAs described in at least two different studies are summarized. Different subsets of T cells (both mouse and human) were activated with either antibodies against CD3 alone (Ab), or together with antibody against CD28 (Abs), or with specific peptides (OVA or gp33-41). Cells were stimulated during different lengths of time ranging from 18 h (18 h) to 7 days (7 d). The studies included in the table are: A (5), B (6), C (7), D (8), E (9), F (10), G (11). Whenever more than one detection method was used, only consistent data obtained with at least two techniques was selected (8). Most studies evaluated miRNA expression with miRNAs arrays, some together with RT-qPCR and Northern Blot, as indicated (x).

The miRNA repertoire changes upon T cell activation (5–11). Figure 1 summarizes miRNA species described to be either upregulated or downregulated upon T cell stimulation. Different studies have yielded data that may appear contradictory, likely due to T cell subset differences, the origin of the sample (murine or human) and the strategy of stimulation. Additional differences stem from the strategy used to evaluate miRNA expression, being arrays the most commonly employed technique, together with RT-qPCR and Northern Blot.

Despite variability, some trends are very consistent, including downregulation of miR-26a, miR-26b, miR-150, miR-181a, miR-223, and miR-342-3p; and upregulation of miR-155 and the miR-17~92 cluster (particularly miR-17-5p, miR-18a-5p, and miR-19b). MiR-146a was downregulated in mouse T cells, but upregulated in human upon activation, while miR-31 behaved in the opposite way, suggesting the existence of species-specific regulatory mechanisms.

In addition to variations in miRNA expression, it would be essential to consider the total abundance of each miRNA in the cell. Interestingly, only 7 miRNAs accounted for around 60% of the total sequencing reads in CD8^+^ T cells (8).

Beyond individual miRNA changes, it is important to highlight that miRNAs undergo a global downregulation upon stimulation. In this regard, almost three times higher total miRNA array hybridization signal has been detected in mouse CD8^+^ naive T cells compared to activated cells (8); similarly, an independent study found a significant downregulation of the total amount of miRNA in stimulated mouse and human CD4^+^ T cells compared to non-stimulated controls (5).

## Lessons from miRNA-deficient models

Dicer is an RNase III endonuclease that controls miRNA biogenesis. It processes precursor miRNA (pre-miRNA) into mature miRNA forms (12–14). Constitutive Dicer KO mice display embryonic lethality (15), indicating the relevance of this enzyme in development. Lineage-specific Dicer-deficient models were therefore required to study the consequences of reduced miRNA function in a tissue-specific manner.

Dicer-deficient CD4^+^ T cells were hyper-responsive to TCR stimulation and produced IL-2 in the absence of co-stimulation (16). After activation, CD4^+^ Dicer-deficient mice showed reduced proliferation, higher levels of apoptosis and a bias towards Th1 differentiation and IFN-γ release (17). In Th1 differentiation, IFN-γ production and a decline in IL-2 secretion occurred earlier in Dicer-deficient than in wild-type CD4^+^ T cells (17). Th2 cells presented reduced levels of GATA3 mRNA and failed to suppress IFN-γ expression (17). Consistently, similar phenotypes were observed in T cells lacking Drosha or its RNA-binding cofactor DGCR8, which form a complex responsible for primary miRNA transcript processing. Drosha-deficient naïve CD4^+^ T cells differentiated into Th1 and Th2, but expressed higher levels of IFN-γ than control cells (18). Similarly, DGCR8-deficient T lymphocytes showed reduced proliferation and an increase in IFN-γ secretion (19). A number of very comprehensive reports have addressed the role of miRNAs in T cell differentiation (20–24). In this review, immunoregulatory molecules responsible for differentiation have been discussed when closely related to T cell activation events.

CD4-specific Dicer deficiency also affects the regulatory T cell compartment, impairing Tregs development in the thymus and reducing their numbers in peripheral lymphoid organs (25). In addition, deficient naïve CD4^+^ T cells activated in the presence of TGF-β expressed significantly less FOXP3 than control cells (25). Besides, several studies have demonstrated that miRNA disruption in Treg cells leads to autoimmune diseases (18, 26, 27).

Dicer-deficient CD8^+^ T lymphocytes responded more rapidly to activation *in vitro*, as indicated by faster CD69 up-regulation and an earlier proliferative response, although their survival was reduced after 2 days (28). CD8^+^ Dicer KO cells also showed a delay in CD69 down-regulation after removal of the TCR-activating stimulus, suggesting a sustained activation of cytotoxic lymphocytes in the absence of miRNAs (28). Furthermore, CD8^+^ Dicer-deficient cells failed to produce an efficient *in vivo* effector response, including lower proliferation and impaired cytokine production (IFN-γ and TNF-α) (28).

**Figure 2:**
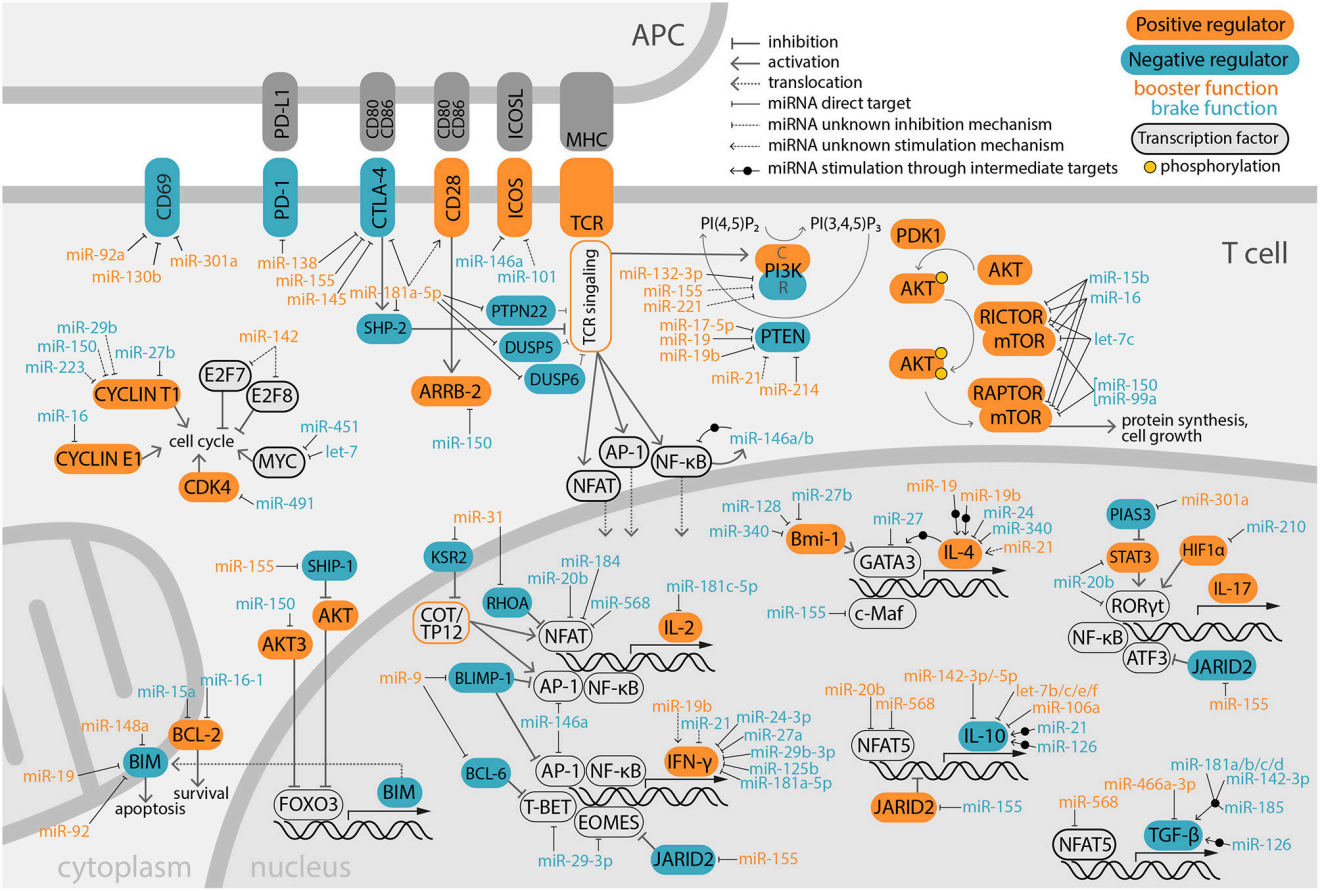
Overview of miRNA modulation on positive and negative immune-regulator molecules. Signaling coming from TCR and costimulatory molecules is integrated by the T lymphocyte promoting cell survival, proliferation and production of effector molecules, such as cytokines. This complex network is fine-tuned by miRNAs that target key immunoregulatory molecules, supporting either T cell activation (booster) or inhibition (brake). MiRNAs exert their function by targeting the mRNA 3′UTR in the cytoplasm, although for simplicity sake some have been depicted in the nucleus, close to their targeted immunoregulators. In PI3K, C and R designated the catalytic and regulatory subunits, respectively.

Models with impaired miRNA synthesis machinery highlight the importance of miRNAs as positive (booster) and/or negative (brake) regulators of T cell development and function, which is a major focus of this review (Figure 2).

MiR-146a mainly acts as a “brake” miRNA, as miR-146a-deficient mice develop chronic inflammation and autoimmunity (29). CD4^+^ and CD8^+^ T cells from miR-146a deficient mice display less apoptosis and increased proliferation, expression of activation markers (CD25 and CD69) and effector cytokines (IL2, IFN-γ, and IL-17A) (30). Likewise, miR-125b is another negative regulator of T cell function, contributing to the maintenance of the naïve state in human CD4^+^ T cells, in which it appears at high levels (31). This effect is at least partly achieved via targeting key molecules for T cell activation, e.g., BLIMP-1, IL-2Rβ, IL-10Rα, and IFN-γ (31). Conversely, other miRNAs boost the immune response. For instance, miR-142-deficient mouse T cells showed reduced proliferation, deregulated cytokine expression and decreased secretion of pro-inflammatory cytokines such as IFN-γ, IL-17, and IL2 in response to activation (32, 33). Other examples of enhancer miRNAs are miR-155 and miR-17~92; miR-155-depleted mice are immunodeficient (34), whereas miR-17~92-deficient T cells exhibited reduced antitumoral responses (35).

## Immunoregulatory molecules as miRNA targets

T cell activation requires that the TCR recognizes a specific antigen bound to the MHC on the surface of an APC in the presence of co-stimulation. PI3K, AKT and mTOR are crucial mediators of T cell activation. Their positive signaling, downstream the TCR, is counter-balanced by negative regulators such as PTEN and BIM. Costimulatory signals are provided by surface receptors expressed on T lymphocytes that interact with specific ligands on APCs, and can be either activating (such as CD28 and ICOS) or inhibitory (like CTLA-4 and PD-1). These activating and inhibitory events are integrated into a net response that triggers the activation and/or repression of transcription factors (NFAT, AP-1, NF-κB, and others). Their nuclear localization promotes the synthesis of immune effector molecules, e.g., cytokines. MiRNAs also control the activation and integration of these pathways to support T cell effector functions while maintaining immune homeostasis. Herein, we review the miRNA-mediated regulation of key molecules involved in T cell activation.

### Cell survival and signaling molecules

#### BIM

The balance between BIM and BCL-2 molecules is essential for the fate of T lymphocytes, and their expression is tightly regulated by miRNAs, promoting either apoptosis or survival. BIM is a pro-apoptotic regulator and tumor suppressor downstream of AKT3, an important mediator of TCR signaling (36, 37). It destabilizes mitochondrial membrane, inducing CASPASE-9 activation and apoptosis. Within the miR-17~92 cluster, miR-19 and miR-92 target BIM 3′UTR mRNA (38). MiR-148a is upregulated in mouse Th1 cells after sustained activation (39). It also targets BIM, promoting cell survival (39). MiR-155 indirectly regulates BIM by targeting SHIP-1, which is a phosphatase that reduces AKT activity (40). In turn, AKT represses FOXO3, which is a transcription factor that promotes BIM expression, thus miR-155 limits BIM expression (40). Conversely, miR-150 promotes apoptosis by downregulating AKT3, which induces the accumulation of BIM (41). Human CD4^+^ T cells with high levels of miR-150 display reduced proliferation, increased apoptosis and lower T cell activation (41).

#### BCL-2

BCL-2 is an anti-apoptotic protein that antagonizes BIM, stabilizing the mitochondrial membrane and preventing its permeabilization (42). Treatment of mice with experimental autoimmune encephalomyelitis with 3,3′-Diindolylmethane (a plant-derived anti-inflammatory compound), induced the upregulation of miR-16 in brain CD4^+^ T cells and suppressed BCL-2; consistently, miR-16 overexpression in mouse CD4^+^ T cells downregulated BCL-2 (43). Interestingly, CD4^+^ T cells from relapsing-remitting multiple sclerosis patients (an autoimmune disease elicited by activated autoreactive T lymphocytes) displayed lower levels of miR-15a and miR-16, correlating with higher levels of their validated target BCL-2 mRNA (44, 45).

#### Cell cycle regulators

Molecules involved in cell cycle progression are essential mediators of T cell proliferation. miR-142-null T cells displayed gross cell cycle alterations, with cells differentially arrested in S and G_2_/M phases (32). Cell-cycle defects were associated to the transcription factors E2F7 and E2F8, which are putative targets for miR-142. MiR-142 is likely responsible of maintaining low levels of both molecules in resting T-cells and limiting their increase upon activation. Treatment of mice with miR-142 antagomir markedly increased survival and reduced clinical symptoms in a murine GVHD model, suggesting a potential new therapeutic strategy (32).

Cyclins are also directly targeted by miRNAs. Several miRNAs (miR-27b, miR-29b, miR-150, and miR-223) promote CYCLIN T1 downregulation in human resting CD4^+^ T cells. The levels of these miRNAs decrease upon activation, correlating with an upregulation of CYCLIN T1 (46). MiR-16 downregulates CYCLIN E1 in mouse CD4^+^ T cells (43). Another molecule involved in cell cycle progression is CDK4, a target of miR-491 in mouse CD8^+^ T cells (47). MYC is a transcription factor involved in cell cycle and proliferation, is targeted by let-7 in mouse CD8^+^ T cells (48) and by miR-451 in both mouse (49) and human (50) CD4^+^ T cells.

#### mTOR

Mammalian Target Of Rapamycin (mTOR) is a metabolic regulator that promotes protein synthesis and cell growth during the onset of T lymphocyte function (51). mTOR kinase and Raptor are part of the complex mTORC1, while mTORC2 includes mTOR and Rictor. Both miR-16 and let-7c target the 3′UTR of mTOR and RICTOR (16). Elevated mTOR activity in Dicer-deficient CD4^+^ T cells and the subsequently increased AKT phosphorylation is associated with a lower activation threshold, overcoming the need of co-stimulation. MiRNA-mediated mTOR down-regulation contributes to the correct discrimination of activating and anergic stimuli and prevents co-stimulation independent IL-2, IFN-γ and TNF-α overproduction (16). mTOR signaling suppression is relevant for Treg induction. In this regard, miR-16 and miR-15b, which are abundantly expressed in Tregs, target RICTOR and mTOR mRNAs (52). Furthermore, miR-150 and miR-99a cooperatively target mTOR, promoting Treg induction (53).

### Co-stimulatory molecules

#### Membrane receptors: ICOS and CD28

Inducible co-stimulatory (ICOS) molecule and CD28 are surface receptors expressed on T cells that recognize specific ligands on APCs, acting as TCR signaling positive regulators (54). In germinal center responses, miR-146a upregulation in Tfh cells downregulates ICOS by interacting with its ligand on germinal center B cells, facilitating the termination of the immune response (55). MiR-101 is highly represented in human naïve CD4^+^ T cells and its transfection into the EL4 murine T cell line downregulates ICOS (56). Regarding CD28, miR-181a-5p overexpression in mouse T cells increases its levels (57), whereas miR-150 limits CD28 co-stimulation by targeting the arrestin β-2 protein (ARRB-2), with a subsequent increase in cAMP levels and inhibition of LCK, PI3K and AKT (58).

#### Cytokines

MiRNA regulation of cytokine expression can be due to direct cytokine mRNA targeting or targeting of transcription factors such as NF-κB, NFAT, or AP-1 or their regulators, often affecting multiple cytokines. For example, miR-146a is induced in mouse CD4^+^ and CD8^+^ T cells upon TCR engagement through NF-κB (30). This miRNA provides negative feedback regulation, downregulating NF-κB by targeting TRAF6 and IRAK1 (30, 59). Compared to wild-type cells, both CD4^+^ and CD8^+^ mouse T cells lacking miR-146a exhibited a higher induction of genes regulated by NF-κB, e.g., BCL-2, CD25, CD69, IL-2, IFN-γ, and IL-17A (30). TRAF6 is also targeted by miR-146b in mouse Tregs (60).

##### IL-2

IL-2 is one of the main signatures of T cell activation. MiRNA-based IL-2 regulation relies on the inhibition of translation by miR-181c-5p (downregulated during T cell activation), which binds to the 3′UTR of IL-2 mRNA (61). It also depends on the miRNA-based downregulation of transcription factors such as NFAT or BLIMP-1. MiR-184 inhibits NFAT1 translation in human CD4^+^ T cells. This is particularly relevant in cells isolated from umbilical cord blood (62). MiR-568 transfection into human CD4^+^ T cells inhibited IL-2 expression after activation, through NFAT5 downregulation (63). MiR-20b also downregulated IL-2 through NFAT5 targeting (64). MiR-31 upregulates IL-2 by inhibiting RHOA, a small GTPase which suppresses NFAT (65, 66). It also targets the kinase suppressor of RAS2 (KSR2), which inhibits the COT/TPI2 signaling pathway (enhancer of IL-2 expression through NFAT and AP-1) (67). MiR-9 (upregulated in activated human CD4^+^ T cells) targets BLIMP-1, de-repressing IL-2 transcription (68). MiR-146a is upregulated around 8 days after stimulation in human CD4^+^ and CD8^+^ T cells, impairing IL-2 production, by targeting AP-1 (69).

##### IFN-γ

IFN-γ release orchestrates Th1 immune responses by activating different cell lineages, e.g., dendritic cells, macrophages or NK cells. MiR-125b maintains T cell naïve state by targeting *IFN-*γ among other genes (31). Several miRNAs repress IFN-γ: miR-24-3p (70) and miR-181a-5p in human CD4^+^ T cells (70, 71); miR-24 and miR-27a in activated human CD8^+^ T cells (72); and miR-29 directly (73) and indirectly, by downregulating T-BET and EOMES, in mouse CD4^+^ T cells (19). On the other hand, miR-19b is required for normal IFN-γ production, restoring IFN-γ expression in miR-17~92-deficient mouse Th1 cells (35). MiR-9 suppresses BLIMP-1 and BCL-6 (repressors of AP-1 and T-BET, respectively), increasing IFN-γ secretion in activated human CD4^+^ T cells (68). Murine miR-21 KO CD4^+^ T cells re-stimulated *in vitro* produced more IFN-γ (74). Moreover, IFN-γ responsiveness is regulated by miR-155, which targets IFN-γRα in activated mouse CD4^+^ T cells, contributing to Th1 differentiation (75).

##### IL-4

T cell activation stimulates the production of IL-4, leading to Th2 responses (76, 77). Its release is controlled directly by miR-24 **[**78] and miR-340 (78), or through the targeting of specific transcription factors and kinases/phosphatases. IL-4 triggers the upregulation of GATA3 dependent STAT6, repressing Th1 differentiation and inducing IL-4 production in a positive feedback loop. Conversely, MiR-27 targets the transcription factor GATA3 (79). BMI1 binds to GATA3, preventing its degradation. CD4^+^ T cells from MS patients display increased expression of miR-27b, miR-128 and miR-340 (78). These miRNAs inhibited Th2 development by targeting BMI1 (78). MiR-155 targets the 3′UTR of c-MAF mRNA, which is another transcription factor involved in IL-4 expression (34). MiR-21 contributes to IL-4 expression, since *in vitro* re-stimulated miR-21-null mouse CD4^+^ T cells produced less IL-4 than wild-type cells (74). Both miR-19a and miR-19b rescued IL-4 production in miR-17~92 cluster-deficient cells by targeting PTEN, SOCS1 and A20 (80).

##### IL-17

TCR signaling promotes expression of the proinflammatory cytokine IL-17 (81–83). IL-17 expression depends on the transcription factor RORγt downstream of STAT3. miR-20b targets both molecules in mouse CD4^+^ T cells (84). RORγt transcription is promoted by HIF-1α, which is targeted by miR-210 (85). In turn, STAT3 is inhibited by the E3 SUMO-protein ligase PIAS3, a target of miR-301a that increases IL-17 secretion (86). MiR-212 targets BCL-6 3′UTR, which is a repressor of Th17 differentiation (87). JARID2, a chromatin-binding protein, recruits the polycomb repressive complex 2 (PRC2) and silences transcription of IL22, IL10, ATF3, TBX21, or EOMES through histone methylation (88). MiR-155 inhibits JARID2, releasing the repression of ATF3, which promotes IL-17 (88). ETS-1, a transcription factor that inhibits Th17 differentiation, is a target of miR-155 (89) and miR-326 (90). Li et al. (91) reported IL-17 downregulation due to IL-23R inhibition by let-7f.

### Inhibitory molecules

#### Membrane receptors: CTLA-4, PD-1, CD69

CTLA-4 and PD-1 are both co-inhibitory receptors that repress TCR signaling via binding to co-stimulators expressed by APCs (54). CTLA-4 (a target of miR-145) is very abundant in human peripheral blood Tregs, in which miR-145 is downregulated (92). MiR-155 also targeted CTLA-4 in mouse (93) and human (94) CD4^+^ T cells. MiR-155 overexpression in human CD4^+^ T cells promoted proliferation, and could underlie chronic inflammation in atopic dermatitis, in which it is highly expressed also by CD4^+^ T cells present in skin lesions (94). MiR-138 targets CTLA-4 and PD-1, promoting tumor-regression by inhibiting tumor-infiltrating Tregs (95). MiR-181a-5p overexpression in mouse T cells decreased CTLA-4 expression, while increasing CD28 levels (57).

CD69 is an early surface marker of lymphocyte activation (96). Dicer KO CD8^+^ T cells up-regulated CD69 more rapidly upon stimulation and retained the expression longer after stimuli removal (28), indicating a potential miRNA-based repression of CD69 in naïve stages that restrains activation. MiR-130b and miR-301a increased their levels during CD8^+^ T cell activation and downregulated CD69 (28). MiR-92, which is downregulated in lamina propria leukocytes from rhesus macaques with chronic simian immunodeficiency virus infection, also targets the 3′UTR of CD69 mRNA (97).

#### Kinases and phosphatases

TCR signaling is mediated by downstream kinases and phosphatases, which undergo a tight regulation that ensures functional activation while avoiding hyperreactivity.

##### PI3K regulatory subunits

Upon TCR and co-receptors engagement, PI3K phosphorylates PI(4,5)P_2_. *PIK3R1* gene encodes the regulatory subunits p85, p50, and p55 (98). MiRNAs upregulated in CD4^+^ activated human T cells, e.g., miR-155 and miR-221 downregulate PIK3R1 (9). MiR-132-3p is upregulated in mouse dendritic cell-activated CD4^+^ T lymphocytes, targeting PIK3R1 mRNA (6).

##### TCR inhibitory phosphatases

Phosphatases downstream the TCR pathway counteract signaling by dephosphorylation. Downregulation of some of these phosphatases by miR-181a-5p generates high levels of phosphorylated intermediates in steady-state (57). MiR-181a-5p targets the phosphatases PTPN22, DUSP5 and DUSP6, which dephosphorylate LCK, ZAP70, and ERK1/2; and SHP-2, which mediates negative costimulatory signals from CTLA-4 (57). Therefore, the expression of this miRNA contributes to reduce the activation threshold, increasing the strength and sensitivity of the T cell to peptides with lower affinity (57). In elderly individuals, reduced expression of miR-181a in CD4^+^ naïve T cells is a cause of the declined T cell responsiveness associated with age (99).

##### PTEN

PTEN dephosphorylates PI(3,4,5)P_3_, antagonizing PI3K. As such, PTEN curbs T cell activation, preserving self-tolerance. Transgenic mice overexpressing miR-17~92 cluster developed lymphoproliferative and autoimmune pathologies associated to the reduced expression of PTEN and BIM (38). PTEN is downregulated by several miRNAs that are increased upon T cell activation: miR-21 (100), miR-214 (7) and the miR-17~92 cluster [miR-17-5p (38), miR-19 (38), and miR-19b (35)]. Consistently, miR-21 and miR-214 expression increased T cell proliferation (7, 100).

#### Cytokines

##### IL-10

IL-10 is an important anti-inflammatory cytokine mainly produced by Th2 and Tregs. It counteracts CD28 signaling and suppresses the expression of IFN-γ and IL-2. IL-10 is directly targeted by miR-142-3p, miR-142-5p (101), miR-let-7e (102), let-7c (103, 104), let-7b (104), let-7f (104), and miR-106a (105). MiRNAs further regulate IL-10 post-transcriptionally by modulating JARID2, NFAT5, p85-β or the programmed cell death protein 4 (PDCD4). JARID2 silences IL-10 and is a target of miR-155, which thus promotes IL-10 expression (88). MiR-568 (downregulated upon human CD4^+^ T cell activation) reduced IL-10 by targeting NFAT5 (63). NFAT5 was also targeted by miR-20b (64). MiR-126 is highly increased after Treg stimulation and promotes IL-10 expression (106), and miR-126 targeting of p85-β and PI3K/AKT pathway modulation is responsible of IL-10 release (106). MiR-21 is upregulated in CD4^+^ T cells from systemic lupus erythematosus patients, and its inhibition led to a decrease in IL-10 production (107). MiR-21 positive regulation of IL-10 secretion likely depends on its targeting of PDCD4, a translation inhibitor (107).

##### TGF-β

TGF-β is expressed in naïve T cells preventing T cell activation until sufficient TCR stimulation downregulates the TGF-β type 1 receptor (108–110). TGF-β induces FOXP3, a key transcription factor that promotes Treg differentiation (111). In addition to IL-10 modulation, miR-568 (63) and miR-126 (106) also regulate TGF-β release. In CD4^+^ mouse T cells from draining lymph nodes, miR-466a-3p (upregulated in mice after skin allograft) targets TGF-β2, limiting Treg generation (112). MiRNAs also regulate TGF-β function at different levels by targeting upstream molecules involved in cytokine production, TGF-β receptors and effector molecules of the TGF-β signaling pathway. GARP is a transmembrane protein specifically expressed in Tregs that cleaves the precursor form of TGF-β1 (113). GARP is targeted by miRNAs which are less abundant in human Tregs than in T helper subsets, e.g., miR-142-3p, miR-185, and miR-181a/b/c/d (113, 114). MiR-17 targets TGFBR2 (TGF-β receptor II) in mouse and human CD4^+^ T cells (35, 115). In addition, it has been found that a set of miRNAs upregulated in naïve CD4^+^ T cells from multiple sclerosis patients target TGFBR1 and/or SMAD4 (both involved in the TGF-β signaling pathway) limiting differentiation into Tregs (116).

## Concluding remarks

MiRNA-mediated modulation of molecules involved in T cell activation remains far from being fully understood, although strides have been made in recent years. There is a need to advance towards a “network study” of miRNA function. Considering more than one miRNA in experimental designs increases its technical complication, but also enables models that simulate the complexity of the physiological scenarios, in which individual miRNAs interact with a set of targets and each target in turn can be regulated by several miRNAs, at different levels, either directly targeting the molecule or indirectly regulating its expression via targeting its receptor and/or transcription factors.

Finally, integrating basic and clinical research (e.g., cancer, autoimmunity, and GVHD) could help to achieve a better understanding of T cell immune-regulation to design new strategies for therapy in T cell related malignancies.

## Author contributions

AR-G wrote the draft manuscript and designed the Figures. LF-M corrected and edited the manuscript. FS-M edited the manuscript. AR-G, LF-M, and FS-M discussed all the items in the manuscript.

### Conflict of interest statement

The authors declare that the research was conducted in the absence of any commercial or financial relationships that could be construed as a potential conflict of interest.

## Abbreviations

AKT3: v-akt murine thymoma viral oncogene homolog 3
APC: antigen-presenting cell
BIM: B-cell lymphoma 2 (Bcl-2) interacting mediator of cell death
CTLA-4: Cytotoxic T lymphocyte-associated antigen 4
GVHD: Graft versus host disease
IL: Interleukin
PD-1: Programmed Death 1
PI(3, 4, 5)P3: phosphatidylinositol-(3,4,5)-triphosphate
PI(4, 5)P2: phosphatidylinositol-(4,5)-biphosphate
PTEN: phosphatase and tensin homolog
TCR: T-cell receptor
Tfh: T follicular helper
TGF-β: Transforming Growth Factor- β
Treg: regulatory T cell
tTreg: Thymic-derived regulatory T cells
UTR: untranslated region.
